# Peripheral sensory function in non‐freezing cold injury patients and matched controls

**DOI:** 10.1113/EP090720

**Published:** 2023-02-20

**Authors:** Jennifer Wright, Heather Massey, Sarah Hollis, Tom Vale, David LH. Bennett, Matthew Maley, Hugh Montgomery, Michael Tipton, Clare Eglin

**Affiliations:** ^1^ Extreme Environments Laboratory, School of Sport, Health and Exercise Science University of Portsmouth Portsmouth UK; ^2^ Regional Occupational Health Team (ROHT) Catterick Catterick Garrison UK; ^3^ Nuffield Department of Clinical Neurosciences University of Oxford UK; ^4^ Environmental Ergonomics Research Centre, Loughborough School of Design and Creative Arts Loughborough University Loughborough UK; ^5^ Department of Medicine University College London London UK

**Keywords:** cold injury, intra epidermal nerve fibre density, neural, pathophysiology, quantitative sensory testing, sensory function, sensory neuropathy

## Abstract

The aim of this study was to compare peripheral sensory neural function of individuals with non‐freezing cold injury (NFCI) with matched controls (without NFCI) with either similar (COLD) or minimal previous cold exposure (CON). Thirteen individuals with chronic NFCI in their feet were matched with the control groups for sex, age, race, fitness, body mass index and foot volume. All undertook quantitative sensory testing (QST) on the foot. Intraepidermal nerve fibre density (IENFD) was assessed 10 cm above the lateral malleolus in nine NFCI and 12 COLD participants. Warm detection threshold was higher at the great toe in NFCI than COLD (NFCI 45.93 (4.71)°C vs. COLD 43.44 (2.72)°C, *P* = 0.046), but was non‐significantly different from CON (CON 43.92 (5.01)°C, *P* = 0.295). Mechanical detection threshold on the dorsum of the foot was higher in NFCI (23.61 (33.59) mN) than in CON (3.83 (3.69) mN, *P* = 0.003), but was non‐significantly different from COLD (10.49 (5.76) mN, *P* > 0.999). Remaining QST measures did not differ significantly between groups. IENFD was lower in NFCI than COLD (NFCI 8.47 (2.36) fibre/mm^2^ vs. COLD 11.93 (4.04) fibre/mm^2^, *P* = 0.020). Elevated warm and mechanical detection thresholds may indicate hyposensitivity to sensory stimuli in the injured foot for individuals with NFCI and may be due to reduced innervation given the reduction in IENFD. Longitudinal studies are required to identify the progression of sensory neuropathy from the formation of injury to its resolution, with appropriate control groups employed.

## Abstract (highlights)

- **What is the central question of this study?**
  Is peripheral sensory function impaired in the chronic phase of non‐freezing cold injury (NFCI)?
- **What is the main finding and its importance?**
  Warm and mechanical detection thresholds are elevated and intraepidermal nerve fibre density is reduced in individuals with NFCI in their feet when compared to matched controls. This indicates impaired sensory function in individuals with NFCI. Interindividual variation was observed in all groups, and therefore a diagnostic cut‐off for NFCI has yet to be established. Longitudinal studies are required to follow NFCI progression from formation to resolution

## 1 INTRODUCTION

Non‐freezing cold injury (NFCI) is caused by prolonged exposure to cold and often wet conditions, and appears to present as a vaso‐neuropathy. In the previous paper (Eglin, Wright, Maley et al., 2023) we investigated the peripheral vascular changes associated with NFCI, whereas in this paper the neural aspects of NFCI are the focus. The neural symptoms and signs of NFCI have long been known, with Ungley and Blackwood (1942) describing sensory disturbances including numbness, tingling, aching and pain, with a delayed response to pinprick stimuli, and an absence of vibration detection at the great toe. More recently, the impact of NFCI on sensory function and intraepidermal nerve fibre density (IENFD) has been investigated in the chronic phase using clinical examination, neurophysiological and quantitative sensory testing (QST) and skin biopsies (Vale et al., 2017).

A standardised protocol for QST has been developed by the German research network of neuropathic pain (DFNS) and includes measures of thermal detection, thermal pain, thermal sensory limen, mechanical detection, mechanical pain and stimulus/response function, wind‐up ratio, vibration detection, and pressure pain (Rolke et al., 2006). These are designed to assess specific afferent nerve fibre types including unmyelinated C fibres responsible for thermal and mechanical pain and thermal detection; myelinated Aβ fibres responsible for touch/vibration detection; and myelinated Aδ fibres responsible for mechanical and thermal pain and cool detection (Koop & Tadi, 2019). The reduced or hypersensitive response to a specific stimulus helps elucidate an individual's sensory profile, and can thereby indicate specific patterns of sensory dysfunction. Significant changes in sensory profile have previously been demonstrated in peripheral neuropathy using the DFNS protocol (Maier et al., 2010). When this QST protocol was applied to participants with chronic NFCI, as assessed in a neuropathy clinic, thresholds for thermal (cold and warm), vibration and mechanical stimuli were all shown to be impaired when compared to normative values derived from a cohort of age‐ and gender‐matched healthy controls in the DFNS database (Vale et al., 2017). In addition, IENFD in calf skin biopsies has been reported to be below the normative range in 86% of those with NFCI, and this reduction in IENFD correlated with increased heat pain thresholds (Vale et al., 2017). These observations are consistent with sensory neuropathy and therefore may be a feature of NFCI. However, these findings are based on comparison with normative values from a cohort of healthy controls derived from the DFNS database for QST and published data for IENFD (Rolke et al., 2006 for QST, and that produced by Lauria, Bakkers, et al., 2010; Lauria, Hsieh, et al., 2010). Where these datasets provide age and sex matching, other crucial characteristics are not accounted for, such as race (Maley et al., 2014), foot surface area (Lunt & Tipton, 2014) and aerobic fitness (Maeda et al., 2005), all of which influence an individual's response to cold and therefore potentially the development of sensory neuropathy in cold injury. Importantly, cold exposure per se (without causing cold injury) may cause long term reduced mechanical, thermal and vibration detection in the extremities (Carlsson et al., 2016). This study sought to compare peripheral sensory function and IENFD in individuals diagnosed with NFCI (NFCI), with matched controls who either had similar levels of previous cold exposure (COLD) or minimal previous cold exposure (CON). It was hypothesised that peripheral sensory function (thermal, mechanical and vibration detection thresholds) would be impaired, and that IENFD would be reduced in NFCI patients when compared to both control groups.

## 2 METHODS

### 2.1 Ethics approval

All participants provided written informed consent and the study received ethical approval from the Ministry of Defence Research Ethics Committee (study reference: 909/MoDREC/18). The study complied with the *Declaration of Helsinki* (1964), as last revised at the 64th World Medical Association General Assembly, Brazil, 2013, except for registration in a database.

### 2.2 Study participants

Seventeen individuals with NFCI were recruited to the study. Of these, three were excluded as they had NFCI in their hands only, and one was unable to complete the full set of tests due to work commitments. Thirteen individuals remained with NFCI in one or both feet. The NFCI group were recruited from a regional UK military cold injuries clinic and did not have a history of frostbite. NFCI diagnosis was based on a detailed history of the estimated degree of cold exposure, symptoms at point of injury, persistence of symptoms and standard primary care level neurological examination, and benchmarked against a standardised set of diagnostic criteria (defined from a mix of animal and human research evidence and case series; see Ministry of Defence, 2021). The conditions and symptoms experienced by the NFCI patients are detailed by Eglin, Wright, Maley et al. (2023). Briefly, the initial injury occurred during military field exercises in freezing conditions (0 to −25°C). Symptoms of the affected area at the time of injury included numbness, pain, paraesthesia, discomfort, swelling and cold. At the time of testing, nine NFCI reported having symptoms from their injury when in normal room temperature including numbness (*n* = 5), paraesthesia (*n* = 1), pain (*n* = 2), cold (*n* = 2) and aching (*n* = 1). Seven NFCI reported that their NFCI negatively affected their quality of sleep due to pain and/or cold in the affected area. Nine NFCI reported experiencing pain in their affected foot/feet in the 24 h prior to the study. Those with Raynaud's phenomenon were excluded using the international consensus criteria for the diagnosis of Raynaud's phenomenon. Assessments were conducted by a medical doctor (S.H.) with over 20 years of experience in reviewing NFCI cases and 7 years of running the defence medical services regional NFCI clinic. At the time of testing, all individuals remained serving members of the British Army.

The 13 NFCI were matched as closely as possible for sex, age, race, estimated aerobic fitness, body mass index (BMI) and foot volume (Table 1) with two control groups. The first control group consisted of cold‐exposed controls (COLD) without a diagnosis of NFCI, who were recruited from UK army soldiers and had therefore been exposed to similar winter military training exercises as the NFCI group. This group was recruited to assess if the response to the tests conducted are caused by cold injury or cold per se. Of 26 COLD participants enrolling in the study, six were unable to complete study testing due to work commitments. Thirteen of the remaining 20 participants were matched as closely as possible to the NFCI patients based on the criteria above (Table 1).

**TABLE 1:** Participant characteristics.

	NFCI (*n* = 13)	COLD (*n* = 13)	CON (*n* = 13)	*P*
Sex	2 female	2 female	2 female	
	11 male	11 male	11 male	
Race	7 White	7 White	7 White	
	6 African/Caribbean	6 African/Caribbean	6 African/Caribbean	
Age (years)	28 (5)	30 (5)	26 (5)	0.1552
Height (cm)	176 (6)	176 (7)	178 (10)	0.075
Mass (kg)	76.5 (6.8)	80.6 (11.7)	80.04 (13.4)	0.346
BMI (kg/m^2^)	24.3 (2.1)	25.7 (3.3)	25.1 (1.7)	0.207
Foot volume (cm^3^)	837 (228)	894 (197)	940 (155)	0.494
Estimated aerobic fitness (ml/min/kg)	68 (8)	69 (11)	59 (11)	0.022^*^

The mean (SD) characteristics of the NFCI group (NFCI), cold‐exposed control group (COLD) and non‐cold‐exposed control group (CON) (*n* = 13 in each group), matched for sex, race, height, mass, BMI, foot volume and estimated aerobic fitness. *P*‐values from ANOVA are presented for the three groups. ^*^Significant differences (*P* ≤ 0.05) were present between two groups. For estimated fitness, COLD > CON (*P* = 0.024).

The second control group comprised civilian participants and it was established through the use of a cold exposure questionnaire (Eglin et al., 2021) that they did not partake in any sports/activities where they were likely to get cold (i.e., they partake in indoor sport/gym activities), and did not report any events of being cold during which they may have sustained a cold injury. Descriptions of the cold exposure experienced by each group are detailed further in Eglin, Wright, Maley et al. (2023). The CON group was included to assess the non‐injured, and non‐cold‐exposed response to the tests conducted. Nineteen CON participants enrolled in the study (no withdrawals) of whom 13 were matched as above.

Participants attended the laboratory wearing T‐shirt and trousers. Their height (SECA 213, SECA, UK), mass (SECA 899, SECA, UK) and foot volume (water displacement method) were measured. Participants then undertook a 6‐min Åstrand–Rhyming submaximal cycle test on a cycle ergometer (Lode Corival CPET, Gronigen, the Netherlands) to provide an estimation of their peak oxygen uptake.

### 2.3 Quantitative sensory testing

The laboratory in which the QST protocol was conducted was quiet and maintained at 24.0 (0.8)°C. The QST protocol was conducted in accordance with the protocol outlined by the German research network for neuropathic pain (DFNS, Rolke et al., 2006). The same researcher (J.W.) conducted the QST assessments. Prior to the commencement of data collection, J.W. undertook QST training with the German research network of neuropathic pain (DFNS) at the University of Mannheim. The only deviation away from the DFNS protocol was to conduct the warm and cool detection and heat and cold pain thresholds a total of five times per participant (rather than three, to allow for familiarisation of the protocol), with the final three values being used in analyses.

The skin temperatures of the lumbar region of the back and the dorsum of the foot were obtained using an infrared camera (FLIR A655sc w/25° lens, 640 × 480 pixels, FLIR Systems, Wilsonville, OR, USA) prior to undertaking the QST protocol. The QST protocol comprised 11 tests which assessed 13 measures of neural function, including warm detection threshold (WDT) and cold detection threshold (CDT), heat pain threshold (HPT) and cold pain threshold (CPT), paradoxical heat sensation (PHS) and thermal sensory limen (TSL) (Somedic modular sensory analyser, Somedic AB, Norra Mellby, Sweden). Mechanical detection threshold (MDT) was assessed using von‐Frey hairs (Optihair2, MRC Systems, Heidelberg, Germany) and mechanical pain threshold (MPT) by pinprick stimuli (MRC Systems, Heidelberg, Germany). Mechanical pain sensitivity (MPS), dynamic mechanical allodynia (DMA) and wind‐up ratio (WUR) were also assessed. Vibration detection threshold (VDT) was obtained using a 64 Hz tuning fork (Granton C 64 Hz Rydel‐Seiffer Tuning Fork, Morton Medical, Cirencester, UK), and pressure pain threshold (PPT) using a pressure algometer (FDN 200, Wagner Instruments, Connecticut, USA). All participants were tested using the same equipment.

Firstly, a control site on the back (positioned to the left of the lumbar spine) was used for assessing detection of thermal, mechanical and mechanical pain stimuli. This was selected as NFCI symptoms are not reported at this location, making it unlikely related neuromuscular injuries will be encountered and an ideal comparison when compared to the peripheral neural system at the foot (Knutti et al., 2014). Following this, warm and cool thermal detection thresholds were conducted on the great toe pad, and then the entire QST protocol was conducted on the dorsum of the foot. This protocol was replicated for all participants, and the QST data were processed in accordance with the guidance provided by the DFNS protocol handbook (Rolke et al., 2006). For each individual dataset, the arithmetic means of the thermal detection threshold, thermal sensory limen, thermal pain threshold, wind‐up ratio, vibration detection threshold, and pressure pain threshold were analysed as the stimuli grading ascended in an equal linear fashion. The geometric mean was used for the mechanical detection threshold, mechanical pain threshold, mechanical pain sensitivity, and dynamic mechanical allodynia as the stimuli grading ascended in a multiplicative fashion. For all measures aside from paradoxical heat sensations, thermal pain thresholds and the vibration detection thresholds, log transformation was undertaken. This processing was in line with the guidelines set by the DFNS protocol handbook (Rolke et al., 2006).

### 2.4 Skin biopsy (IENFD)

Twenty‐two participants (9 NFCI and 13 COLD) volunteered for a skin biopsy to assess intraepidermal nerve fibre density (IENFD) allowing analysis between six NFCI and six COLD participants who were matched for sex, race, age, estimated aerobic fitness, BMI and foot volume. A 3 mm skin punch biopsy was obtained 10 cm above the lateral malleolus in line with the methods described by Vale et al. (2017), and the subsequent IENFD analysis was undertaken by a qualified and experienced researcher (T.V.) at the Nuffield Department of Clinical Neurosciences, University of Oxford. All samples were obtained and analysed in accordance with the guidelines described by the European Federation of Neurological Societies/Peripheral Nerve Society (Lauria, Bakkers, et al., 2010; Lauria, Hsieh, et al., 2010), as described in detail in Vale et al. (2017).

### 2.5 Data analysis

Data presented within this study are available at pure.port@ac.uk. For each QST variable, D'Agostino–Pearson and Shapiro–Wilk tests for normality were conducted. The three participant groups were compared using one‐way ANOVA of parametric data (with Bonferroni adjustment for multiple comparisons), or a Kruskall–Wallis test of non‐parametric data (with Dunn's adjustment for multiple comparisons). The IENFD for the NFCI and COLD groups was compared using Welch's *t*‐test with both matched and unmatched participants. Analysis was conducted using GraphPad Prism software (version 8.2.1; GraphPad Software Inc., San Diego, CA, USA).

Further analysis of the QST data was completed using eQUISTA software (version 1.3.5). This converted QST data to *Z*‐scores, which compared the data to normative values produced by the DFNS of a population who were considered to be uninjured. For the great toe and foot data, this population consisted of White volunteers who participated across 10 assessment centres, which included 110 females and 70 males with an mean (SD) age of 38.4 (12.9) years (female 38.9 (13) years, male 37.5 (13) years; Magerl et al., 2010; Rolke et al., 2006). The normative trunk values were based on 162 healthy participants aged between 18 and 79 years (Pfau et al., 2014). *Z*‐scores which fall 2 standard deviations outside of the normative range are indicative of a loss or gain in function (Rolke et al., 2006).

Spearman's correlation analysis was conducted for all neural, vascular, and biomarker measures (including QST and IENFD) to establish if links between measures were present. Further details of this analysis can be found in the first paper of this series (Eglin, Wright, Maley et al., 2023).

Owing to the low sample size of this study, *post hoc* power analysis was conducted in G*Power (version 3.1.9.7). The power (1 − β) values are presented for all tests conducted at each location for the QST and skin biopsy. For the QST tests *post hoc* analysis, the effect size (*f*) was set at 0.25, and for the IENFD *post hoc* analysis the effect size (*d*) was set at 0.5. The level of significance was set at 0.05 for both QST tests and IENFD. Power (1 − β) values equal to or above 0.8 were considered the threshold at which a true effect was observed.

## 3 RESULTS

The laboratory in which the protocol was conducted was maintained at 24.0 (0.8)°C, and temperature did not differ between groups (NFCI, 23.7 (0.6)°C; COLD, 23.8 (0.7)°C; CON, 24.0 (0.5)°C; *F =* 0.694 *P* = 0.500). The skin temperature of the lumbar spine region and dorsum of the foot was similar between groups (back: NFCI, 32.9 (1.1)°C; COLD 32.1 (1.2)°C; CON, 32.2 (1.0)°C; *F =* 2.023, *P* = 0.145; foot: NFCI, 28.2 (2.5)°C; COLD, 29.3 (2.9)°C; CON, 30.6 (2.5)°C; *F =* 2.509, *P* = 0.094).

### 3.1 Quantitative sensory testing

#### 3.1.1 Back

WDT, MDT and MPT were not significantly different between groups (WDT, *F =* 0.838, *P* = 0.441; MDT, *F =* 1.857, *P* = 0.171; MPT, *F =* 1.636, *P* = 0.209; Table 2). CDT was lower in NFCI (*F =* 10.250, *P* = 0.002) and COLD (*P* = 0.001) when compared to CON (Table 2).

**TABLE 2:** Quantitative sensory testing.

	NFCI		COLD		CON		
Test	Mean (SD)	% CV	Mean (SD)	% CV	Mean (SD)	% CV	*P*
Back							
Warm detection threshold (°C)	2.97 (0.72)	24.24	3.42 (0.94)	27.49	4.23 (3.43)	81.09	0.441
Cold detection threshold (°C)	1.80 (0.96)^*^	53.33	1.65 (0.49)^*^	29.70	4.23 (3.34)^*,**^	78.96	**0.001**
Mechanical detection threshold (mN)	19.15 (26.42)	137.96	11.32 (14.17)	125.18	5.78 (4.67)	80.80	0.171
Mechanical pain threshold (mN)	40.41 (35.26)	87.26	36.97 (23.50)	63.57	73.34 (75.45)	102.88	0.209
Foot							
Warm detection threshold (°C)	8.11 (3.75)	46.24	8.16(3.00)^**^	36.76	6.09 (2.85)^**^	46.80	**0.033**
Cold detection threshold (°C)	3.84 (1.92)	50.00	3.85 (2.20)	57.14	2.86 (1.60)	55.94	0.142
Thermal sensory limen (°C)	4.94 (3.06)	61.94	4.42 (3.58)	81.00	2.87 (1.97)	68.64	0.127
Paradoxical heat sensation	0.38 (1.02)	268.42	0.69 (1.58)	228.99	0.66 (1.5)	227.27	0.870
Heat pain threshold (°C)	47.07 (2.72)	5.78	47.95 (2.18)^**^	4.55	43.75 (4.10)^**^	9.37	**0.008**
Cold pain threshold (°C)	18.33 (8.84)	48.23	16.67 (8.07)	48.41	20.21 (8.24)	40.77	0.270
Mechanical detection threshold (mN)	23.61 (33.59)^*^	142.27	10.49 (5.76)^**^	54.91	3.83 (3.69)^*,**^	96.34	**0.001**
Mechanical pain threshold (mN)	46.19 (48.78)	105.61	49.86 (46.27)	92.80	77.95 (92.27)	118.37	0.628
Mechanical pain sensitivity (mN)	6.52 (13.35)	204.75	4.48 (6.55)	146.21	5.46 (8.75)	160.26	0.961
Dynamic mechanical allodynia (mN)	0.21 (0.57)	271.43	0.43 (0.97)	225.58	0.33 (0.98)	296.97	0.974
Wind‐up ratio	2.83 (2.09)	73.85	3.24 (3.46)	106.79	2.83 (1.97)	69.61	0.773
Vibration threshold	6.99 (1.41)	20.17	7.06 (1.07)	15.16	7.35 (0.86)	11.70	0.674
Pressure pain threshold (kPa)	4.97 (1.89)	38.03	5.24 (1.48)	28.24	5.40 (2.23)	41.30	0.886
Toe							
Warm detection threshold (°C)	14.39 (4.34)^*^	30.16	11.26 (2.58)^*^	22.91	11.41 (5.08)	44.52	**0.048**
Cold detection threshold (°C)	8.44 (4.15)	49.17	7.03 (3.45)	49.08	6.01 (3.10)	51.58	0.648

The mean (SD) and percentage confidence intervals (CV) for quantitative sensory testing (QST) parameters conducted on the lumbar region of the back (Back), dorsum of the foot (Foot) and plantar surface of the great toe (Toe) in the NFCI, COLD and CON groups (*n* = 13 in each group). *P*‐value from ANOVA or Kruskal–Wallis test are presented for the three groups. ^*^ and ^**^ indicate where significant differences (*P* ≤ 0.05) were present between two groups. Back CDT: NFCI < COLD and CON (COLD *P* = 0.002, CON *P* = 0.001). Foot WDT: COLD > CON (*P* = 0.040); HPT: COLD > CON (*P* = 0.008); MDT: NFCI > CON (*P* = 0.001), COLD > CON (*P* = 0.002). Toe WDT: NFCI > COLD (*P* = 0.046). COLD, cold‐exposed controls; CON, non‐cold‐exposed controls; NFCI, individuals with NFCI.

#### 3.1.2 Dorsum of the foot

WDT was higher in COLD than CON participants (*F =* 3.652, *P* = 0.040), but NFCI was similar to both COLD (*P* > 0.999) and CON (*P* = 0.134). No differences were present between groups for CDT (*F =* 2.037, *P* = 0.142). HPT was higher in COLD than CON participants (*H* (3) = 9.708, *P* = 0.006), but the NFCI group was not different from either COLD (*P* = 0.824) or CON (*P* = 0.152). MDT was significantly higher in NFCI (*F* = 8.683, *P* = 0.001) than CON (*P* = 0.003), and COLD was significantly higher than CON (*P* = 0.002). However, no difference was observed between NFCI and COLD (*P* > 0.999, Table 2). There were no between‐group differences for MPT (*F =* 0.470, *P* = 0.628), WUR (*H* (3) = 0.515, *P* = 0.773), VDT (*H* (3) = 0.790, *P* = 0.674), PPT (*F =* 0.121, *P* = 0.886), TSL (*H* (3) = 4.121, *P* = 0.127), PHS (*H* (3) = 0.278, *P* = 0.870), MPS (*F =* 0.039, *P* = 0.961) or DMA (*H* (3) = 0.053, *P* = 0.974; Table 2).

#### 3.1.3 Great toe plantar surface

WDT was higher in NFCI than COLD (*H* (3) = 6.086, *P* = 0.046), but was not different from CON (*P* = 0.295). There were no differences in WDT between COLD and CON (*P* > 0.999, Table 2). CDT did not differ between groups (*H* (3) = 0.869, *P* = 0.648, Table 2).

**FIGURE 1:**
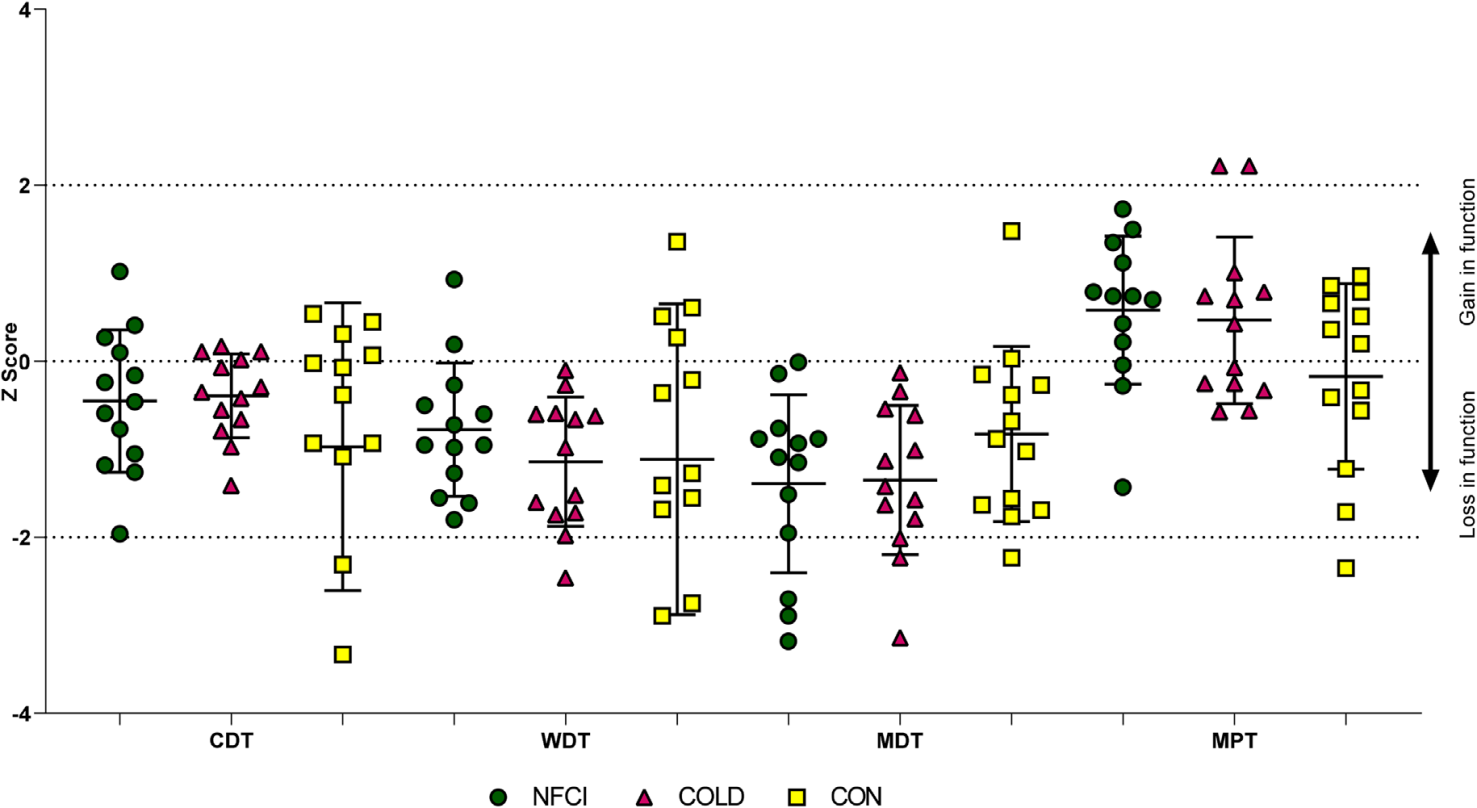
*Z*‐scores from quantitative sensory testing – lumbar region. *Z*‐scores for cold detection threshold (CDT), warm detection threshold (WDT), mechanical detection threshold (MDT) and mechanical pain threshold (MPT) at the lumbar region of the back. Individual data and mean (SD) presented for NFCI (green, *n* = 13), COLD (magenta, *n* = 13) and CON (yellow, *n* = 13) groups. A *Z*‐score >2 indicates significantly increased sensitivity, whereas a *Z*‐score < −2 indicates significantly decreased sensitivity. No further statistical analyses were conducted. COLD, cold‐exposed controls; CON, non‐cold‐exposed controls; NFCI, individuals with NFCI.

**FIGURE 2:**
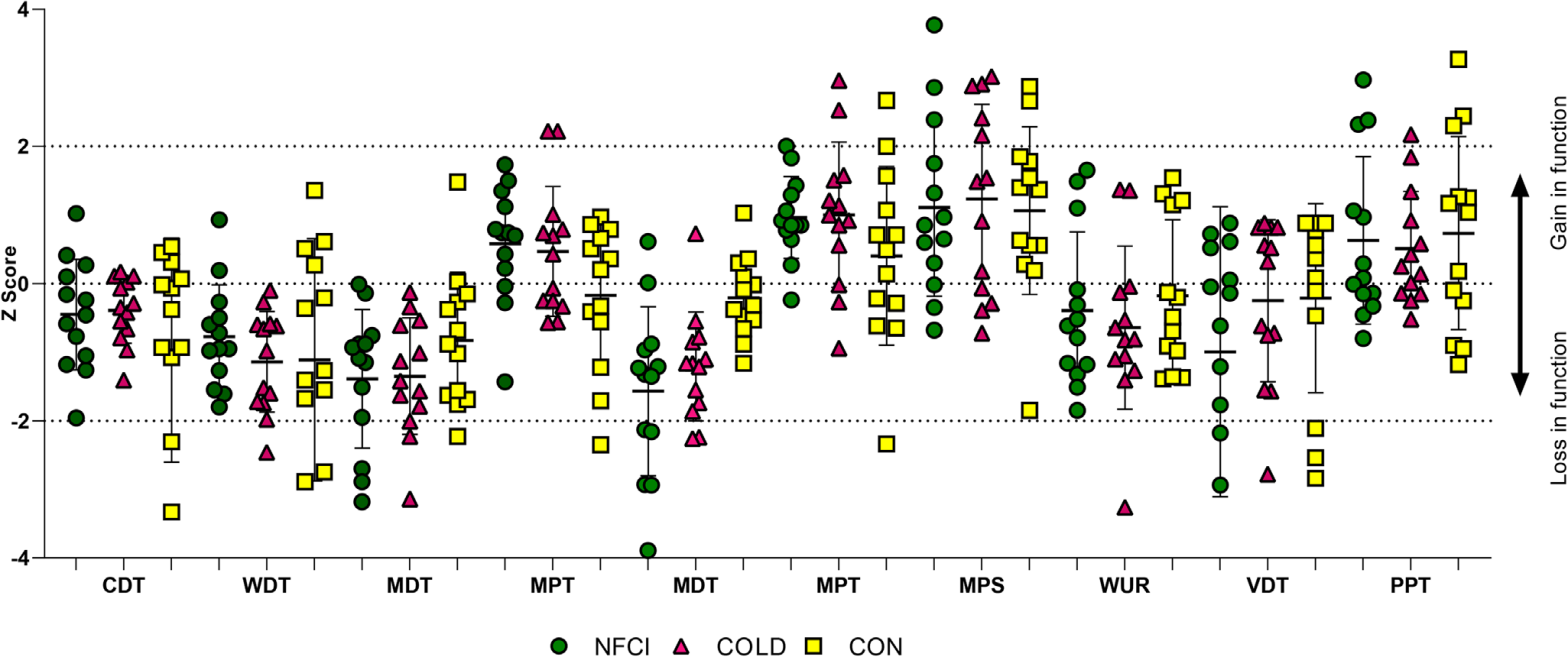
*Z*‐scores from quantitative sensory testing – foot. *Z*‐scores for cold detection threshold (CDT), warm detection threshold (WDT), thermal sensory limen (TSL), cold pain threshold (CPT), heat pain threshold (HPT), mechanical detection threshold (MDT), mechanical pain threshold (MPT), mechanical pain sensitivity (MPS), wind‐up ratio (WUR), vibration detection threshold (VDT) and pressure pain threshold (PPT) at the dorsum of the foot. Individual data with mean (SD) are presented for NFCI (green, *n* = 13), COLD (magenta, *n* = 13) and CON (yellow, *n* = 13) groups. A *Z*‐scores >2 indicates significantly increased sensitivity, whereas a *Z*‐score <−2 indicates significantly decreased sensitivity. No further statistical analyses were conducted. COLD, cold‐exposed controls; CON, non‐cold‐exposed controls; NFCI, individuals with NFCI.

A similar prevalence of *Z*‐scores 2 standard deviations outside of the normative range (according to the DFNS normative data within eQUISTA) was seen for each group in all tests (Figures 1 and 2).

### 3.2 Skin biopsy

**FIGURE 3:**
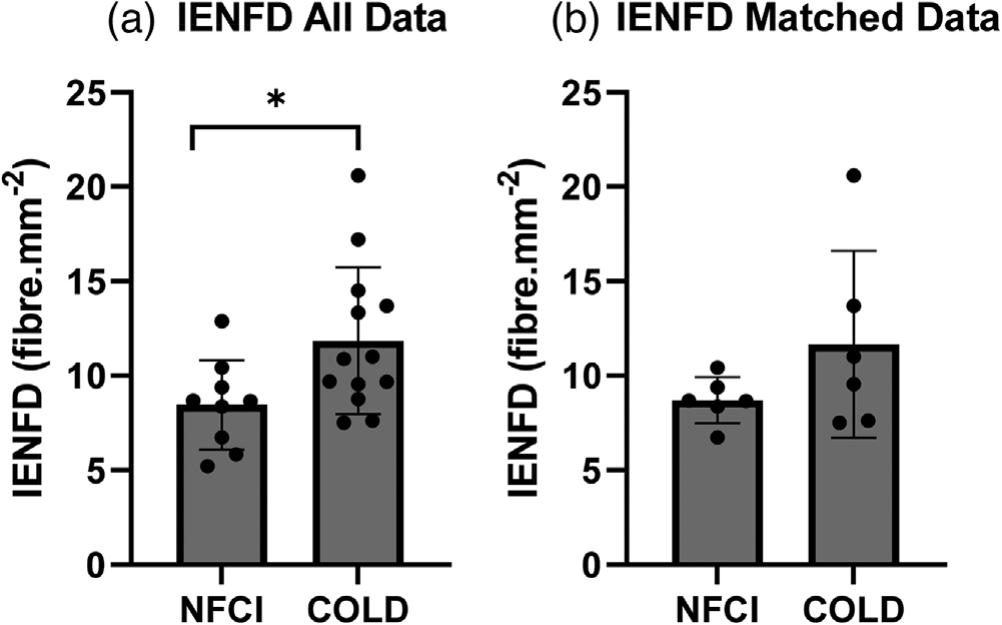
Intra‐epidermal nerve fibre density. The intra‐epidermal nerve fibre density (IENFD) from calf skin biopsies in NFCI and COLD groups. Individual data points (dots), mean (bar) and standard deviation (line) of all the data are shown in (a) (NFCI: *n* = 9; and COLD: *n* = 13); and for matched participants in (b) (both groups *n* = 6) *P = 0.020 for IENFD between NFCI and COLD (all data). Welch's *t*‐test indicated no difference between matched NFCI and COLD (*P* = 0.210). COLD, cold‐exposed controls; NFCI, individuals with NFCI.

IENFD was significantly lower (*F* = 2.69, *P* = 0.020, Figure 3a) in NFCI (*n* = 9) compared to COLD (*n* = 13) when all data were analysed. The coefficient of variation of IENFD was 27.9% versus 32.7% in NFCI vs COLD, respectively (unmatched data). When NFCI and COLD participants were matched for race, sex and physical characteristics (*n* = 6), no difference between NFCI and COLD was observed (*t* = 1.423, *P* = 0.210, coefficient of variation 14.0% vs. 42.4%, respectively; Figure 3b).

### 3.3 *Post hoc* power analysis

None of the tests which formed the QST, nor IENFD, reached the power (1 − β) threshold of 0.8 (Table 3).

**TABLE 3:** Post‐hoc power analysis.

Test	Power (1 − β)
Back	
Warm detection threshold (°C)	0.12
Cold detection threshold (°C)	0.79
Mechanical detection threshold (mN)	0.67
Mechanical pain threshold (mN)	0.32
Foot	
Warm detection threshold (°C)	0.28
Cold detection threshold (°C)	0.15
Thermal sensory limen, (°C)	0.18
Paradoxical heat sensation	0.19
Heat pain threshold (°C)	0.76
Cold pain threshold (°C)	0.12
Mechanical detection threshold (mN)	0.13
Mechanical pain threshold (mN)	0.08
Mechanical pain sensitivity (mN)	0.06
Dynamic mechanical allodynia (mN)	0.05
Wind‐up ratio	0.06
Vibration threshold	0.24
Pressure pain threshold (kPa)	0.09
Toe	
Warm detection threshold (°C)	0.23
Cold detection threshold (°C)	0.14
Calf	
Matched IENFD	0.12
All data IENFD	0.20

## 4 DISCUSSION

This is the first study to compare sensory function and IENFD in individuals with NFCI in their feet with two matched control groups either with (COLD) or without (CON) similar previous exposure to cold. The main finding was that the warm detection threshold at the great toe and mechanical detection threshold at the dorsum of the foot were both higher in NFCI, indicating a loss of function. IENFD was reduced in NFCI compared to COLD when data were unmatched, but not when they were matched (likely due to the low sample size). Therefore, the impaired sensory function in warm and mechanical detection may be linked to the reduced IENFD in NFCI compared to COLD. The cold detection threshold was lower in the NFCI and COLD groups compared to CON at the lumbar region of the back, but no statistically significant difference was observed at the foot (despite cold detection at the foot being nearly 1°C higher in NFCI and COLD compared to CON; and that at the great toe being 2.4°C higher in NFCI, and 1°C higher in COLD than CON). This may be attributed to the variability within the groups tested. As such, an increased cold detection threshold may be due to altered central mediation of cold stimuli stemming from cold exposure, but this is not possible to confirm within this study. Finally, cold exposure in the absence of a cold injury diagnosis may result in decreased mechanical and heat pain sensitivity, as the COLD group had a higher mechanical and heat pain detection threshold when compared to the CON group. It must be acknowledged that measures of QST and IENFD are not fully powered within this study (Table 3), and therefore provide only a preliminary insight of how IENFD and sensory function may be impacted by NFCI in the chronic phase.

The ability to detect increasing temperature (warm detection threshold) at the great toe was impaired in the NFCI group when compared to COLD, with the mean temperature difference detected in NFCI being 14.4 (4.3)°C and 11.3 (2.6)°C in COLD. The primary nerve fibre types responsible for the transmission of warm detection stimuli in the peripheral nervous system are the thinly myelinated (Aδ) and unmyelinated (C) nerve fibres (Cecil et al., 2012). In this instance, where the warm detection threshold is greater in NFCI, it would be reasonable to infer a level of damage within the Aδ and C nerve fibres, causing impaired transmission of thermal stimuli to the central nervous system. This may present as thermal hypoesthesia in NFCI, with a reduced ability to detect increasing thermal stimuli within a certain range.

An increased warm detection threshold was observed in the great toe but not the dorsum of the foot in individuals with NFCI. This could be due to NFCI affecting more distal regions of the extremities, since these regions will experience greater cooling during injurious cold exposure (Castellani & Young, 2016; Yamazaki, 2015). In addition, as nerve fibre density in healthy non‐glabrous skin sites (such as the great toe pad) is higher than in glabrous skin sites (such as the dorsum of the foot; Arthur & Shelley, 1959), non‐glabrous sites may be more susceptible to NFCI following periods of ischemia.

The mechanical detection threshold refers to the threshold force at which punctate stimuli (in the form of von Frey hairs) are detected upon application to the skin surface, and is mediated through myelinated Aβ nerve fibres (Koga et al., 2005). On the dorsum of the foot, this was higher in NFCI participants (Table 2), perhaps indicating reduced transmission within the Aβ fibres.

A previous study identified abnormal thresholds for cold and warm detection, vibration and mechanical detection in NFCI individuals when compared to normative values (Vale et al., 2017). The findings from QST and IENFD in this study somewhat support the work of Vale et al. (2017) in that abnormal warm and mechanical detection and IENFD have been seen across both studies. A greater degree of sensory neuropathy (with both cold detection and vibration detection thresholds also being abnormal) may be present in the study by Vale et al. (2017), as many individuals were likely to have been at the more severe end of the chronic NFCI phenotype having been recruited from a neuropathy clinic. This is supported by the responses to the Douleur Neuropathique en 4 (DN4) pain questionnaire (Bouhassira et al., 2005) presented in both studies, in which NFCI individuals averaged a higher score of 7.5 in the study by Vale et al. (2017) versus 5.0 in the current study (Eglin, Wright, Maley et al., 2023). A second possible cause of the greater impairments in sensory function noted by Vale et al. (2017) may be that QST assessments were compared to normative values produced by the DFNS rather than to data from matched control groups as presented in the current study.

Unfortunately, due to the invasive nature of obtaining a skin biopsy, the uptake amongst participants in this study was low (NFCI *n* = 9 and COLD *n* = 12) and was further reduced when participants were matched (*n* = 6). Although underpowered (Table 3), the results from all the data in the current study indicate a decline in IENFD may occur with NFCI (Figure 3a) supporting previous findings by Vale et al. (2017). However, when just matched NFCI and COLD participants were compared (*n* = 6, Figure 3b), no significant difference in IENFD was observed. This could be due to the small sample size, or it could be due to a potential overlap between the NFCI and COLD group. Whilst every effort was made to recruit control participants who did not have a cold injury, it is possible that some of the participants in the COLD group had NFCI but were unaware of it, or were not disclosing it due to the negative effect it might have on their careers. A reduction in IENFD without any changes in thermal or mechanical detection thresholds has been reported in a single case study following severe cold exposure indicating there may be an effect of cold per se (Krøigård et al., 2018). This highlights the importance of using a combination of measures to inform a diagnosis of NFCI.

In the current study, no correlations were observed between IENFD and the QST measures. In contrast, Vale et al. (2017) reported a negative correlation between IENFD and heat pain thresholds, which may reflect their participants’ more severe injury and greater sample size (*n* = 27, NFCI). IENFD was, however, found to correlate with measures of vascular function reported in the previous paper (Eglin, Wright, Maley et al., 2023) with slow rates of rewarming being associated with decreased IENFD, indicating NFCI is a vaso‐neuropathy.

Vibration detection, heat pain and cold pain thresholds have been reported to be abnormal in frostbitten regions when compared to control participants (Carlsson et al., 2014). In contrast, none of these QST components were different from the control groups in our study, perhaps due to the different pattern of injury between freezing and NFCI, although further research is required. Interestingly in this study, no differences in cold thermal detection and cold pain thresholds were present between groups. In the cold exposure questionnaire described in the previous paper (Eglin, Wright, Maley et al., 2023), individuals with NFCI rated themselves as being worse than average at coping with the cold than either COLD or CON. This indicates that their perception may be based on alterations in their vascular response to cold exposure (as discussed in Eglin, Wright, Maley et al., 2023) which result in colder skin, rather than alterations in thermal detection thresholds as assessed during QST. Thus, whilst the pain associated with NFCI is likely to be neural in origin, the intolerance to cold may be vascular in nature. Therefore, in order to characterise the changes associated with NFCI, both neural and vascular assessments must be undertaken concomitantly.

As with other environmentally induced clinical conditions, considerable data variability is observed between individuals with NFCI (Tipton et al., 2021). This variability is likely caused by differences in circumstances encountered when acquiring the injury (i.e., environmental temperature, duration of cold exposure, clothing worn, and if any mitigation strategies were in place to prevent the worsening of NFCI when suspected); variation in individual susceptibility to or response to cold exposure (Haman et al., 2022); and variation in the inherently subjective nature of sensory perception, which will vary depending on multiple intra‐ and interindividual cognitive factors. Given the variability seen within the control groups, the pre‐injury status (i.e., their history of previous cold exposure) may also affect the risk of cold injury and its severity. This variability was observed when *Z*‐scores were calculated from the normative DFNS database (Magerl et al., 2010; Rolke et al., 2006). Whilst the majority of the 13 participants in each group fell within normal parameters, a similar number of NFCI and control participants fell outside of the normal range at both the control site (lumbar region of the lower back) and dorsum of the foot (Figures 1 and 2).

There are benefits and disadvantages to using the normative values from the eQUISTA software, with which the QST data from the NFCI and control groups were compared. Although it provides a sizable database collected using a standardised methodology, the eQUISTA database consists of predominantly White females over a 5‐decade age range, with other confounding variables such as height, mass, BMI, foot volume and estimated fitness not detailed (Lunt & Tipton, 2014; Maeda et al., 2005; Maley et al., 2014; Rivner et al., 2001; Smolander, 2002). Consequently, the eQUISTA software may not contain a representative dataset from which to compare the NFCI patients presented as part of this study – mainly young men, half of whom were African/Caribbean (Table 1). The different demographics of our control groups might also explain why a similar number of CON compared to NFCI had *Z*‐scores outside the 2 standard deviations of the eQUISTA normative data despite being screened prior to participation. Similarly, Üçeyler et al. (2018) undertook QST on 273 healthy participants (155 females, 118 males, aged 17–89 years) and also reported *Z*‐scores greater than 2 standard deviations from the control values when compared to the eQUISTA database. Therefore, the eQUISTA database may be beneficial for use in studies which are unable to recruit their own control population(s); in the current study a direct comparison of NFCI with COLD and CON control groups is thought to be a more valid, despite the smaller sample size. With such variability both between and within individuals, however, it is not recommended that QST is used on its own to diagnose impaired sensory function in NFCI. Instead it is suggested it be used in combination with other forms of neural assessment such as IENFD, and considered in context of a history of relevant exposure, clinical symptoms and sensory signs on clinical examination.

### 4.1 Conclusion

This is the first study to investigate peripheral sensory function using QST and measure IENFD in individuals with NFCI compared to matched control groups. The use of matched control groups overcame the potential limitations of using normative data from an unknown population, and also accounted for the effect of cold exposure per se. The results suggest that NFCI is associated with a hyposensitivity to warm and mechanical stimuli in the affected region, as well as a likely reduction in IENFD. Differences between the results of this study and previous research (Vale et al., 2017) may reflect differences in the severity of NFCI and the individuals’ injury progression as well as individual variability. Therefore, a longitudinal study with appropriate controls spanning from the injurious cold exposure to recovery is required, alongside the production of a NFCI severity scale which is reflective of the modern phenotype of NFCI.

## AUTHOR CONTRIBUTIONS

This research was conducted in laboratories at the Infantry Training Centre Catterick, the Royal Military Academy Sandhurst, and the University of Portsmouth. Each author contributed the following: Jennifer Wright, Hugh Montgomery, Michael Tipton and Clare Eglin: conception or design of the work, acquisition or analysis or interpretation of data for the work, and drafting the work or revising it critically for important intellectual content. Heather Massey, Tom Vale, Matthew Maley: acquisition or analysis or interpretation of data for the work, and drafting the work or revising it critically for important intellectual content. Sarah Hollis: conception or design of the work, and drafting the work or revising it critically for important intellectual content. All authors have approved the final version of the manuscript, and agree to be accountable for all aspects of the work in ensuring that questions related to the accuracy or integrity of any part of the work are appropriately investigated and resolved. All persons designated as authors qualify for authorship, and all those who qualify for authorship are listed.

## CONFLICT OF INTEREST

None of the authors listed within this paper has any conflicts of interests.

## Supporting information

Statistical Summary Document

## Data Availability

The data that support the findings of this study are openly available in pure at pure.port@ac.uk; https://doi.org/10.17029/8b2a6b87-b193-4b3f-a7ee-fea5f4e05275.

## References

[eph13304-bib-0001] Arthur, R. P. , & Shelley, W. B. (1959). The innervation of human epidermis. Journal of Investigative Dermatology, 32(3), 397–411. 13641817 10.1038/jid.1959.69

[eph13304-bib-0002] Bouhassira, D. , Attal, N. , Alchaar, H. , Boureau, F. , Brochet, B. , Bruxelle, J. , Cunin, G. , Fermanian, J. , Ginies, P. , Grun‐Overdyking, A. , Jafari‐Schluep, H. , Lantéri‐Minet, M. , Laurent, B. , Mick, G. , Serrie, A. , Valade, D. , & Vicaut, E. (2005). Comparison of pain syndromes associated with nervous or somatic lesions and development of a new neuropathic pain diagnostic questionnaire (DN4). Pain, 114(1), 29–36. 15733628 10.1016/j.pain.2004.12.010

[eph13304-bib-0003] Carlsson, D. , Burström, L. , Heldestad Lilliesköld, V. , Nilsson, T. , Nordh, E. , & Wahlström, J. (2014). Neurosensory sequelae assessed by thermal and vibrotactile perception thresholds after local cold injury. International Journal of Circumpolar Health, 73(1), 23540. 10.3402/ijch.v73.23540 PMC3929118 24624368

[eph13304-bib-0004] Carlsson, D. , Pettersson, H. , Burström, L. , Nilsson, T. , & Wahlström, J. (2016). Neurosensory and vascular function after 14 months of military training comprising cold winter conditions. Scandinavian Journal of Work, Environment & Health, 42(1), 61–70. 10.5271/sjweh.3530 26473467

[eph13304-bib-0005] Castellani, J. W. , & Young, A. J. (2016). Human physiological responses to cold exposure: Acute responses and acclimatization to prolonged exposure. Autonomic Neuroscience, 196, 63–74. 26924539 10.1016/j.autneu.2016.02.009

[eph13304-bib-0006] Cecil, R. L. F. , Goldman, L. , & Schafer, A. I. (2012). Goldman's Cecil Medicine, Expert Consult Premium Edition–Enhanced Online Features and Print, Single Volume, 24: Goldman's Cecil Medicine (Vol. 1). Elsevier Health Sciences.

[eph13304-bib-0045] Eglin, C. M. , Costello, J. T. , Tipton, M. J. , & Massey, H. (2021). Previous recreational cold exposure does not alter endothelial function or sensory thermal thresholds in the hands or feet. Experimental Physiology, 106(1), 328–337. 32394510 10.1113/EP088555

[eph13304-bib-0011] Eglin, C. M. , Wright, J. A. , Maley, M. , Hollis, S. , Massey, H. , Montgomery, H. , & Tipton, M. J. (2023). The peripheral vascular responses in non‐freezing cold injury and matched controls. Experimental Physiology, 108(3), 420–437. 36807667 10.1113/EP090721 PMC10103892

[eph13304-bib-0016] Haman, F. , Souza, S. C. , Castellani, J. W. , Dupuis, M. P. , Friedl, K. E. , Sullivan‐Kwantes, W. , & Kingma, B. R. (2022). Human vulnerability and variability in the cold: Establishing individual risks for cold weather injuries. Temperature, 9(2), 158–195. 10.1080/23328940.2022.2044740 PMC9467591 36106152

[eph13304-bib-0017] Knutti, I. A. , Suter, M. R. , & Opsommer, E. (2014). Test–retest reliability of thermal quantitative sensory testing on two sites within the L5 dermatome of the lumbar spine and lower extremity. Neuroscience Letters, 579, 157–162. 25064700 10.1016/j.neulet.2014.07.023

[eph13304-bib-0018] Koga, K. , Furue, H. , Rashid, M. H. , Takaki, A. , Katafuchi, T. , & Yoshimura, M. (2005). Selective activation of primary afferent fibers evaluated by sine‐wave electrical stimulation. Molecular Pain, 1(1), 1744–8069–1–13. 10.1186/1744-8069-1-13 PMC1083421 15813963

[eph13304-bib-0019] Koop, L. K. , & Tadi, P. (2019). Neuroanatomy, sensory nerves. StatPearls. https://europepmc.org/article/nbk/nbk539846 30969668

[eph13304-bib-0020] Krøigård, T. , Wirenfeldt, M. , Svendsen, T. K. , & Sindrup, S. H. (2018). Asymptomatic loss of intraepidermal nerve fibers with preserved thermal detection thresholds after repeated exposure to severe cold. Brain and Behavior, 8(3), e009147. 29541548 10.1002/brb3.917 PMC5840450

[eph13304-bib-0021] Lauria, G. , Bakkers, M. , Schmitz, C. , Lombardi, R. , Penza, P. , Devigili, G. , Smith, A. G. , Hsieh, S.‐T. , Mellgren, S. I. , Umapathi, T. , Ziegler, D. , Faber, C. G. , & Merkies, I. S. (2010a). Intraepidermal nerve fiber density at the distal leg: A worldwide normative reference study. Journal of the Peripheral Nervous System, 15(3), 202–207. 21040142 10.1111/j.1529-8027.2010.00271.x

[eph13304-bib-0022] Lauria, G. , Hsieh, S. T. , Johansson, O. , Kennedy, W. R. , Leger, J. M. , Mellgren, S. I. , Nolano, M. , Merkies, I. S. J. , Polydefkis, M. , Smith, A. G. , Sommer, C. , Valls‐Solé, J. , & European Federation of Neurological Societies; Peripheral Nerve Society . (2010b). European Federation of Neurological Societies/Peripheral Nerve Society Guideline on the use of skin biopsy in the diagnosis of small fiber neuropathy. Report of a joint task force of the European Federation of Neurological Societies and the Peripheral Nerve Society. European Journal of Neurology, 17(7), 903–e49. 20642627 10.1111/j.1468-1331.2010.03023.x

[eph13304-bib-0023] Lunt, H. , & Tipton, M. (2014). Differences in conductive foot cooling: A comparison between males and females. European Journal of Applied Physiology, 114, 2635–2644. 25173096 10.1007/s00421-014-2988-5

[eph13304-bib-0024] Maeda, T. , Sugawara, A. , Fukushima, T. , Higuchi, S. , & Ishibashi, K. (2005). Effects of lifestyle, body composition, and physical fitness on cold tolerance in humans. Journal of Physiological Anthropology and Applied Human Science, 24(4), 439–443. 16079594 10.2114/jpa.24.439

[eph13304-bib-0025] Magerl, W. , Krumova, E. K. , Baron, R. , Tölle, T. , Treede, R. D. , & Maier, C. (2010). Reference data for quantitative sensory testing (QST): Refined stratification for age and a novel method for statistical comparison of group data. Pain, 151(3), 598–605. 20965658 10.1016/j.pain.2010.07.026

[eph13304-bib-0026] Maier, C. , Baron, R. , Tölle, T. R. , Binder, A. , Birbaumer, N. , Birklein, F. , Gierthmühlen, J. , Flor, H. , Geber, C. , Huge, V. , Krumova, E. K. , Landwehrmeyer, G. B. , Magerl, W. , Maihöfner, C. , Richter, H. , Rolke, R. , Scherens, A. , Schwarz, A. , Sommer, C. , … Treede, R. D. (2010). Quantitative sensory testing in the German Research Network on Neuropathic Pain (DFNS): Somatosensory abnormalities in 1236 patients with different neuropathic pain syndromes. Pain, 150(3), 439–450. 20627413 10.1016/j.pain.2010.05.002

[eph13304-bib-0027] Maley, M. J. , Eglin, C. M. , House, J. R. , & Tipton, M. J. (2014). The effect of ethnicity on the vascular responses to cold exposure of the extremities. European Journal of Applied Physiology, 114(11), 2369–2379. 25081130 10.1007/s00421-014-2962-2

[eph13304-bib-0028] Ministry of Defence (2021). JSP 539: Heat illness and cold injury: Medical management. Part 2 guidance. London, MoD 2021.

[eph13304-bib-0031] Pfau, D. B. , Krumova, E. K. , Treede, R. D. , Baron, R. , Toelle, T. , Birklein, F. , Eich, W. , Geber, C. , Gerhardt, A. , Weiss, T. , Magerl, W. , & Maier, C. (2014). Quantitative sensory testing in the German Research Network on Neuropathic Pain (DFNS): Reference data for the trunk and application in patients with chronic postherpetic neuralgia. Pain, 155(5), 1002–1015. 24525274 10.1016/j.pain.2014.02.004

[eph13304-bib-0033] Rivner, M. H. , Swift, T. R. , & Malik, K. (2001). Influence of age and height on nerve conduction. Muscle & Nerve, 24(9), 1134–1141. 11494265 10.1002/mus.1124

[eph13304-bib-0034] Rolke, R. , Baron, R. , Maier, C. A. , Tölle, T. R. , Treede, R. D. , Beyer, A. , Binder, A. , Birbaumer, N. , Birklein, F. , Bötefür, I. C. , Braune, S. , Flor, H. , Huge, V. , Klug, R. , Landwehrmeyer, G. B. , Magerl, W. , Maihöfner, C. , Rolko, C. , Schaub, C. , … Wasserka, B. (2006). Quantitative sensory testing in the German Research Network on Neuropathic Pain (DFNS): Standardized protocol and reference values. Pain, 123(3), 231–243. 16697110 10.1016/j.pain.2006.01.041

[eph13304-bib-0035] Smolander, J. (2002). Effect of cold exposure on older humans. International Journal of Sports Medicine, 23(2), 86–92. 11842354 10.1055/s-2002-20137

[eph13304-bib-0036] Tipton, M. J. , Corbett, J. , Eglin, C. M. , Mekjavic, I. B. , & Montgomery, H. (2021). In pursuit of the unicorn. Experimental Physiology, 106(2), 385–388. 33174651 10.1113/EP089147

[eph13304-bib-0037] Üçeyler, N. , Vollert, J. , Broll, B. , Riediger, N. , Langjahr, M. , Saffer, N. , Schubert, A.‐L. , Siedler, G. , & Sommer, C. (2018). Sensory profiles and skin innervation of patients with painful and painless neuropathies. Pain, 159(9), 1867–1876. 29863528 10.1097/j.pain.0000000000001287

[eph13304-bib-0038] Ungley, C. C. , & Blackwood, W. (1942). Peripheral vasoneuropathy after chilling immersion foot and immersion hand. The Lancet, 240(6216), 447–451.

[eph13304-bib-0039] University of Portsmouth . (2022, December). Pure . pure.port@ac.uk.

[eph13304-bib-0040] Vale, T. A. , Symmonds, M. , Polydefkis, M. , Byrnes, K. , Rice, A. S. , Themistocleous, A. C. , & Bennett, D. L. (2017). Chronic non‐freezing cold injury results in neuropathic pain due to a sensory neuropathy. Brain, 140(10), 2557–2569. 28969380 10.1093/brain/awx215 PMC5841153

[eph13304-bib-0042] Yamazaki, F. (2015). The cutaneous vasoconstrictor response in lower extremities during whole‐body and local skin cooling in young women with a cold constitution. Journal of Physiological Sciences, 65(5), 397–405. 10.1007/s12576-015-0378-3 PMC10717359 25850923

